# Premature Graying as a Consequence of Compromised Antioxidant Activity in Hair Bulb Melanocytes and Their Precursors

**DOI:** 10.1371/journal.pone.0093589

**Published:** 2014-04-02

**Authors:** Ying Shi, Long-Fei Luo, Xiao-Ming Liu, Qiong Zhou, Shi-Zheng Xu, Tie-Chi Lei

**Affiliations:** Department of Dermatology, Renmin Hospital of Wuhan University, Wuhan, Hubei Province, China; University of Tennessee, United States of America

## Abstract

Intricate coordinated mechanisms that govern the synchrony of hair growth and melanin synthesis remain largely unclear. These two events can be uncoupled in prematurely gray hair, probably due to oxidative insults that lead to the death of oxidative stress-sensitive melanocytes. In this study, we examined the gene expression profiles of middle (bulge) and lower (hair bulb) segments that had been micro-dissected from unpigmented and from normally pigmented hair follicles from the same donors using quantitative real-time RT-PCR (qPCR) arrays. We found a significant down-regulation of melanogenesis-related genes (TYR, TYRP1, MITF, PAX3, POMC) in unpigmented hair bulbs and of marker genes typical for melanocyte precursor cells (PAX3, SOX10, DCT) in unpigmented mid-segments compared with their pigmented analogues. qPCR, western blotting and spin trapping assays revealed that catalase protein expression and hydroxyl radical scavenging activities are strongly repressed in unpigmented hair follicles. These data provide the first clear evidence that compromised antioxidant activity in gray hair follicles simultaneously affects mature hair bulb melanocytes and their immature precursor cells in the bulge region.

## Introduction

A recent worldwide survey showed that 74% of people between the ages of 45 and 65 have grey hair, and that occurs earliest in people of Caucasian descent, followed by Asians and Africans [1]. Hair is considered to grey prematurely only if it occurs before the age of 20 years in Whites, before 25 years in Asians and before 30 years in Africans [2]. Prematurely graying hair (also termed canities) imposes a psychosocial burden on sufferers since it is often regarded as a visible sign of rapidly progressing old age, ill health and bodily decline [2]–[4]. In spite of the fact that the onset of hair graying is genetically controlled and inheritable, there is very little known about the mechanism(s) by which functional melanocytes are lost from anagen graying hair follicles [1], [2]. Emerging evidence shows that reactive oxygen species (ROS) accumulate in human gray/white scalp hair follicles up to millimolar concentrations, which likely causes oxidative damage to hair follicle melanocytes [5], [6].

Mature melanocytes are densely distributed in hair bulbs to sustain active melanogenesis that is strictly coupled to the anagen stage of the hair cycle [7]–[11]. Thus far, the precise mechanism(s) governing the synchrony of hair growth and melanin synthesis has remained largely unclear. Isolation and short-term co-culture of primary keratinocytes, melanocytes and dermal papilla fibroblasts derived from human scalp skin tissues are common strategies to dissect the regulation of anagen-coupled melanogenesis [12]–[14]. Unfortunately, in vitro co-culture studies with established cell lines or primary cell cultures could have led to artificial outcomes and some inaccuracies in earlier studies since hair follicles are composed of several types of cells that span the range of differentiation states, for which it is considered a dynamic miniorgan [15]. Graying hair offers a unique opportunity to study the uncoupling of melanin production with growth of the hair shaft [8]. Although deficient antioxidant activity was reported in human graying hair follicles [5], [6], it remains to be determined whether an impaired antioxidant defense in gray hair follicles simultaneously affects mature hair bulb melanocytes and their immature precursor cells in the bulge region, which would have a critical implication for restoring pigmentation to the affected gray hair.

In this study, we micro-dissected hair bulbs and mid-segments (corresponding to the bulge region) from unpigmented and from pigmented hair follicles isolated from the same human donors. The expression levels of genes encoding characteristic markers for mature melanocytes, melanocyte stem cells and keratinocyte stem cells in the hair bulbs and mid-segments were analyzed using quantitative real-time PCR (qRT-PCR) arrays and the anti-oxidative properties of these segmented hair follicle tissues was investigated in parallel using a range of techniques [16]. The results demonstrate that both mature hair bulb melanocytes and immature melanocyte precursor cells in the bulge region of gray hair follicles are depleted, at least to some extent, and those effects that could be ascribed to reduced levels of catalase protein and activity.

## Materials and Methods

### 1. Patient Recruitment and Isolation of Whole Anagen Hair Follicles

**Figure 1:**
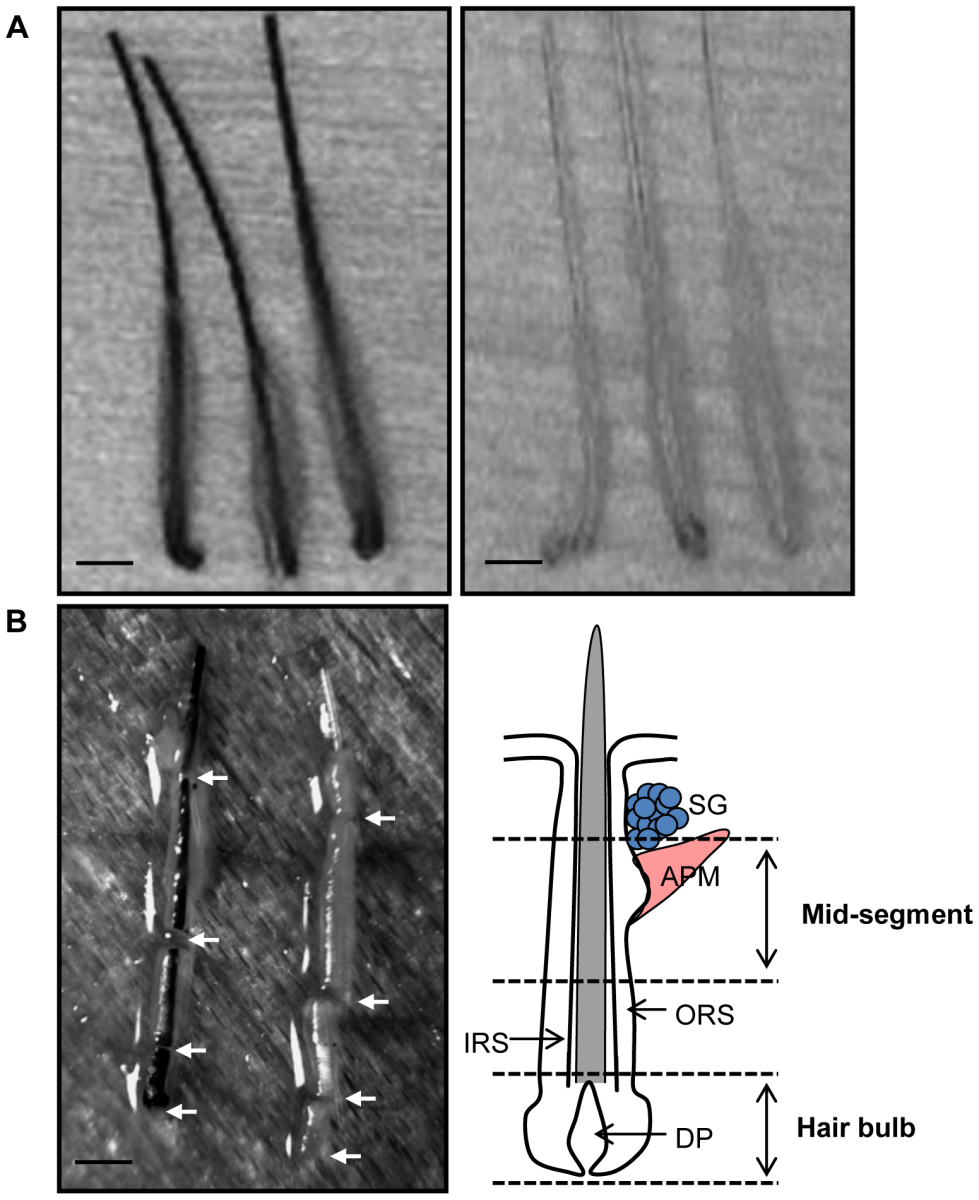
Hair bulbs and mid-segments of hair follicles were micro-dissected from human scalp skin tissues. (A) Pigmented (left) and unpigmented (right) whole anagenic hair follicles; (B) Each hair follicle was further excised using an ophthalmic scalpel under a stereoscope to harvest hair bulbs and mid-segments, as indicated by the arrows (left). Schematic view (right) showing the histological structures corresponding to hair bulbs and mid-segments of hair follicles. Scale bar = 1 mm.

This study was carried out on 9 Chinese patients under 25 years of age who suffered from premature gray hair (Table 1). Written informed consent was obtained from each participant before enrollment. The Ethical Committee of the Renmin Hospital of Wuhan University approved this study and supervised its compliance with the Declaration of Helsinki Guidelines. Pigmented and unpigmented hair follicles were individually extracted from the scalp using a micromotor-driven skin punch device (Mecicamat S.A., Malakoff, France) [17], [18]. Each hair follicle was further excised using an ophthalmic scalpel under a stereoscope to harvest the hair bulbs and mid-segments (corresponding to the bulge region) (Figure 1A), which were then used for subsequent isolation of RNA and protein extraction.

**Table 1:** Clinical and demographic characteristics of patients in this study.

Case No.	Sex (F/M)	Age	Diagnosis*	Surgical procedure	Biopsy site
1	F	20	Canities+ Scar	Scar removal	Occipitalia
2	M	22	Canities+ AA	Hair transplantation	Occipitalia
3	M	25	Canities+ AA	Hair transplantation	Occipitalia
4	M	23	Canities+ AA	Hair transplantation	Occipitalia
5	M	21	Canities+ AA	Hair transplantation	Occipitalia
6	M	20	Canities+ AA	Hair transplantation	Occipitalia
7	M	24	Canities+ AA	Hair transplantation	Occipitalia
8	M	25	Canities	Volunteer	Occipitalia
9	M	23	Canities	Volunteer	Tempus

*AA: Androgenic alopecia.

### 2. Fenton Reaction and Hydroxyl Radical Measurement using a Spin Trapping Assay

The effects of hair bulbs and mid-segments from pigmented and from unpigmented hair follicles on hydroxyl radical (•OH) generation in the Fenton reaction were studied using a spin trapping method, according to our previous report [19]. FeSO_4_ was dissolved in distilled water, while all other solutions were dissolved in 0.1 M phosphate buffer (pH 7.4). The spin trap compound 5,5-dimethyl-1-pyrroline-N-oxide (DMPO) was purchased from Sigma Chemical Co. (St. Louis, MO. Catalog# D5766). Each reaction was carried out in a total of 50 μl in an Eppendorf tube containing 260 mM H_2_O_2_, 0.4 mM FeSO_4_, 400 mM DMPO and identical amounts of minced tissue samples as noted. In the control, metal-free water was substituted for the sample. The Fenton reaction was initiated by the addition of H_2_O_2_, then 50 μl of the reaction mixture was placed in an ESR quartz flat cell. Exactly 20 s after the addition of H_2_O_2_, the ESR spectra of the DMPO-•OH spin adducts were recorded at room temperature using a Bruker ER 200D-SRC ESR spectrometer (Bruker Analytische Messtechnik GmbH, Rheinstetten, Germany) operating at 9.53 GHz microwave frequency, 20 mW microwave power, 100 kHz modulation frequency, and 0.05 mT modulation amplitude. •OH scavenging activity was calculated using the equation: •OH scavenging activity = [1−(H/H_0_)]×100%, in which H and H_0_ represent relative peak height (amplitude) of the second peak of the DMPO-•OH spin adduct with or without sample, respectively.

### 3. RNA Extraction and qRT- PCR Arrays

Thirty to 50 hair bulbs and mid-segments of isolated hair follicles were dissected from the same patients using a micro-dissecting protocol [20] and were pooled in a RNase-free Eppendorf tube containing 20 μL TRIzol reagent (Invitrogen, Eugene, OR, USA). The tissues were then homogenized with a micro-homogenizer (Kimble, Toledo, OH, USA). Total RNAs were extracted from tissues derived from pigmented and from unpigmented hair follicles using TRIzol reagent according to the instructions of the manufacturer and were quantified by measuring absorbance (A value) at 260 nm. A total of 1 μg RNA for each sample was used for reverse transcription using an RT-PCR Kit (Catalog#CTB101; CT Biosciences, China) on an ABI 9700 Thermocycler (ABI, Foster City, CA). PCR arrays were performed with customized PCR containing pre-dispensed primers (CT Biosciences, China) on a LightCycler 480 (Roche Diagnostics, Mannheim, Germany) using SYBR MasterMix (Catalog#CTB101; CT Biosciences, China). Each PCR assay contained 10 ng of synthesized cDNA. The thermocycler parameters were performed with an initial denaturation at 95°C for 5 min followed by 45 cycles of denaturation at 95°C for 10 sec, annealing at 60°C for 10 sec and extension at 72°C for 10 sec. Relative changes in gene expression were calculated using theΔΔCt (threshold cycle) method. Housekeeping genes such as B2M, ACTB, GAPDH, RPL27, HPRT1 and OAZ1 were used to normalize the amounts of RNA. Fold change values were calculated using the formula of 2^–ΔΔCt^. The amplification products of 3 panels of genes associated with melanogenic enzymes, anti-oxidative enzymes and specific marker genes for stem cells or precursors of melanocytes and keratinocytes (Table 2) were also confirmed by visualization of ethidium bromide-stained DNA after agarose gel electrophoresis [20].

**Table 2:** Primers used in RT-PCR assays.

Gene symbol	Accession number	Primer sequence
**ACTB**	NM_001101.3	Forward: 5′-AGCGAGCATCCCCCAAAGTT-3′
		Reverse: 5′-GGGCACGAAGGCTCATCATT-3′
**CAT**	NM_001752.3	Forward: 5′-GATGTGCATGCAGGACAATCAG-3′
		Reverse: 5′-GCTTCTCAGCATTGTACTTGTCC-3′
**GPX1**	NM_000581.2	Forward: 5′-ACGATGTTGCCTGGAACTTT-3′
		Reverse: 5′-TCGATGTCAATGGTCTGGAA-3′
**SOD1**	NM_000454.4	Forward: 5′-AGGGCATCATCAATTTCGAG-3′
		Reverse: 5′-TGCCTCTCTTCATCCTTTGG-3′
**TYR**	NM_000372	Forward: 5′-CAGCTTTCAGGCAGAGGTTC-3′
		Reverse: 5′-GCTTCATGGGCAAAATCAAT-3′
**DCT**	NM_001129889	Forward: 5′-AGTGATTCGGCAGAACATCC-3′
		Reverse: 5′-AGTTCCAGTAGGGCAAAGCA-3′
**TYRP1**	NM_000550.2	Forward: 5′-GCAGAATGAGTGCTCCTAAACTCC-3′
		Reverse: 5′-CCTGATGATGAGCCACAGCG-3′
**MITF-M**	NM_198178.2	Forward: 5′-TTATAGTACCTTCTCTTTGCC-3′
		Reverse: 5′-GCTTGCTGTATGTGGTACTTG-3′
**MC1R**	NM_002386.3	Forward: 5′-GCAGCAGCTGGACAATGTCA-3′
		Reverse: 5′-GCCCCAGCAGAGGAAGAAAA-3′
**KRT15**	NM_002275.3	Forward: 5′-GAGAACTCACTGGCCGAGAC-3′
		Reverse: 5′-CTGAAGAGGCTTCCCTGATG-3′
**KRT19**	NM_002276.4	Forward: 5′-TTTGAGACGGAACAGGCTCT-3′
		Reverse: 5′-AATCCACCTCCACACTGACC-3′
**TGFB1**	NM_000660.4	Forward: 5′-GCCCTGGACACCAACTATTGCT-3′
		Reverse: 5′-AGGCTCCAAATGTAGGGGCAGG-3′

### 4. Western Blot Analysis

The tissue samples were washed in PBS and lysed in extraction buffer containing 1% Nonidet P-40, 0.01% SDS and a protease inhibitor cocktail (Roche, Indianapolis, IN, USA). Protein contents were determined with a BCA assay kit (Pierce, Rockford, IL, USA) and equal amounts of each protein extract (10 μg per lane) were resolved using 10% SDS polyacrylamide gel electrophoresis (SDS-PAGE). Following transblotting to Immobilon-P membranes (Millipore, Bedford, MA, USA) and blocking with 5% nonfat milk in saline buffer, the membranes were incubated with anti-catalase antibody (Abcam, Cambridge, MA, USA) at a 1∶1000 dilution, washed with PBS-T, and then were incubated with horseradish peroxidase-conjugated anti-rabbit IgG (Amersham, Piscataway, NJ, USA) at a dilution of 1∶10,000. Immunoreactive bands were detected by enhanced chemiluminescence using an ECL kit (Amersham, Piscataway, NJ, USA). Immunoblotting of β-actin served as a loading control.

### 5. Catalase Activity Analysis

Catalase activity was determined spectrophotometrically using a commercial catalase analysis kit (Beyotime Biotechnology Co., Nanjing, China), as described previously [21]. Briefly, tissue extracts were treated with excess H_2_O_2_ to decompose catalase for specific times as noted in the text, after which the remaining H_2_O_2_ coupled with a substrate was treated with peroxidase to generate a red product, N-4-antipyryl-3-chloro-5-sulfonate-p-benzoquinone monoimine, which absorbed maximally at 520 nm. The H_2_O_2_ consumption per min was converted to units of enzymatic activity on the basis of a standard curve obtained testing scalar units of bovine catalase. Units were corrected for the protein content of each tissue extract.

### 6. Statistical Analyses

All data are expressed as means ± standard deviation (SD). Differences between two groups were determined using the two-tailed Student *t*-test. *P*<0.05 is considered to be statistically significant. All statistical analyses were performed using GraphPad Prism (Ver. 5) (GraphPad Software, San Diego, CA, USA).

## Results

### 1. Gene Expression Profiles in Hair Bulbs and in Mid-segments of Hair Follicles

**Figure 2:**
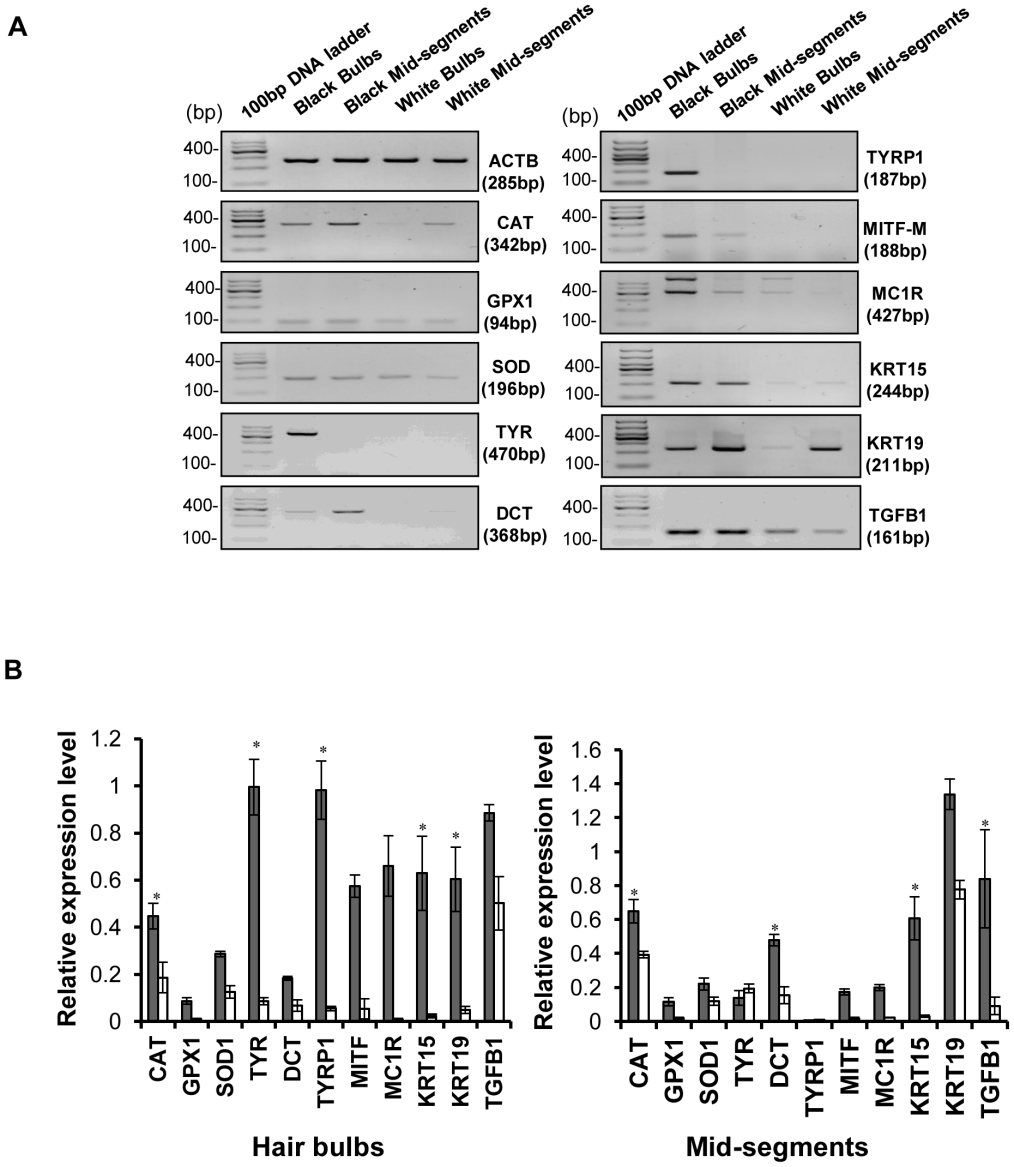
Gene expression profiles of isolated hair follicles analyzed by semi-quantitative RT-PCR. (A) Total RNA was extracted from a pool of 30–50 hair bulbs and mid-segments of hair follicle tissues. RT-PCR amplification was performed using primers specific for the molecular signature genes of the mature hair bulb melanocytes, the immature precursor cells of melanocytes, and anti-oxidant enzymes etc. as indicated in the gels and in Table 2. RT-PCR products were analyzed by electrophoresis on 1.0% agarose gels and images of PCR products are presented in reversed black and white in which the DNA band is black. PCR product sizes for each set of primers are noted in parentheses and were determined by comparison with a 100-bp DNA ladder (far left lane of each panel). (B) The intensity of each band was quantified using Image J densitometry software (NIH, Bethesda, MD, USA). The relative expression level of each targeted gene was normalized to expression of the housekeeping gene β-actin and is reported as relative expression from 3 independent experiments. *P<0.05.

We first analyzed the gene expression patterns typical for mature melanocytes (TYR, TYRP1, MITF), melanocyte precursor cells (DCT, KIT, PAX3), keratinocyte stem cells (KRT15, KRT19) and antioxidant enzymes (CAT, SOD, GPX1) in the hair bulbs and mid-segments of unpigmented (white) and of pigmented (black) hair follicles using qRT-PCR arrays, as summarized in Table 3. A panel of genes encoding melanogenesis-related genes (TYR, TYRP1, MITF, PAX3, POMC, KIT, SOX10) in unpigmented hair bulbs was suppressed more than 20-fold compared with pigmented hair bulbs. Putative marker genes of melanocyte precursor cells (PAX3, SOX10, DCT) were markedly decreased in unpigmented mid-segments compared with pigmented mid-segments. These findings showed that functional melanocytes in the hair bulbs and immature melanocyte precursor cells in the bulge region were depleted in gray hair. The expression level of genes encoding antioxidant enzymes (except SOD2) was significantly decreased in unpigmented hair bulbs compared with pigmented hair bulbs, especially catalase, which was reduced 44-fold in unpigmented hair bulbs and 18-fold in unpigmented mid-segments relative to the pigmented analogues. To confirm the array data, we selected one or two genes from each category and performed RT-PCR analyses. Similar molecular changes in the gray hair follicles were found by semi-quantitative RT-PCR, as shown in Figure 2. Interestingly, a significant down-regulation of marker genes typical of keratinocyte stem cells (KRT15 and KRT19) and up-regulation (>50-fold) of the P53 gene was discerned in unpigmented mid-segments, which might indicate the existence of activated keratinocyte stem cells [22] and alternative P53-mediated antioxidation [23].

**Table 3:** Fold changes of gene expression in unpigmented hair follicles compared with pigmented hair follicles (qRT-PCR array analysis was performed on one subject).

Gene symbol (full name)	Gene ID	Fold change (W vs. B)	
		Hair bulbs	Mid-segments
**Melanogenesis-related genes**			
**DCT (** ***dopachrome tautomerase*** **)**	1638	↓ 5.59	↓ 14.12
**TYR (** ***tyrosinase*** **)**	7299	↓ 45.95	↓ 109.14
**TYRP1 (** ***tyrosinase-related protein 1*** **)**	7306	↓125.53	↓ 54.95
**MITF (** ***microphthalmia-associated transcription factor*** **)**	4286	↓ 52.42	↓ 1.80
**PAX3 (** ***paired box 3*** **)**	5077	↓ 21.74	↓ 16.68
**POMC (** ***proopiomelanocortin*** **)**	5443	↓104.84	↓ 2.99
**ASIP (** ***agouti signal protein*** **)**	434	↓ 9.66	↑ 1.042
**KIT (** ***proto-oncogene c-kit*** **)**	3815	↓ 30.74	↑ 16.68
**SOX10 (** ***SRY-box containing gene 10*** **)**	6663	↓125.53	↓ 1.01
**Antioxidant enzyme genes**			
**CAT (** ***catalase*** **)**	847	↓ 44.08	↓ 18.25
**SOD1 (** ***superoxide dismutase 1, soluble*** **)**	6647	↑ 4.88	↑ 1.80
**SOD2 (** ***Mn superoxide dismu*** **tase)**	6648	↑ 5.425	↑ 1.46
**GPX1 (** ***glutathione peroxidase 1*** **)**	2876	↓ 1.88	↑ 1.35
**MSRA (** ***methionine sulfoxide reductase A*** **)**	4482	↓ 11.33	↓ 1.30
**MSRB1 (** ***methionine sulfoxide reductase B1*** **)**	51734	↓ 2.50	↓ 1.57
**Putative marker genes for stem cell and niche**			
**KRT15 (** ***keratin 15*** **)**	3866	↓ 20.87	↓ 9.18
**KRT19 ** ***(keratin 19*** **)**	3880	↓ 25.17	↓ 5.17
**TNC (** ***tenascin C*** **)**	3371	↓ 10.94	↓ 5.43
**ITGB1 (** ***integrin beta 1*** **)**	3688	↑ 5.49	↑ 1.80
**CD200 (** ***CD200 molecule*** **)**	4345	↑ 1.17	↑ 1.79
**LGR5 (** ***leucine-rich repeat containing G protein-coupled receptor 5*** **)**	8549	↓ 5.51	↑ 5.06
**TGFB1 ** ***(transforming growth factor beta1*** **)**	7040	↓18.05	↓ 5.57
**ITGA8 (** ***integrin alpha 8*** **)**	8516	↓ 17.53	↑ 1.02
**NPNT (** ***nephronectin*** **)**	255743	↓ 12.5	↓ 7.26
**TP53 (** ***tumor P53*** **)**	7157	↑ 3.41	↑ 50.91

*↑: up-regulation; ↓: down = regulation; W: unpigmented hair follicle (HF); B: pigmented HF.

### 2. Suppression of Catalase Protein and Catalytic Activity in Hair Bulbs and in Mid-segments of Hair Follicles

**Figure 3:**
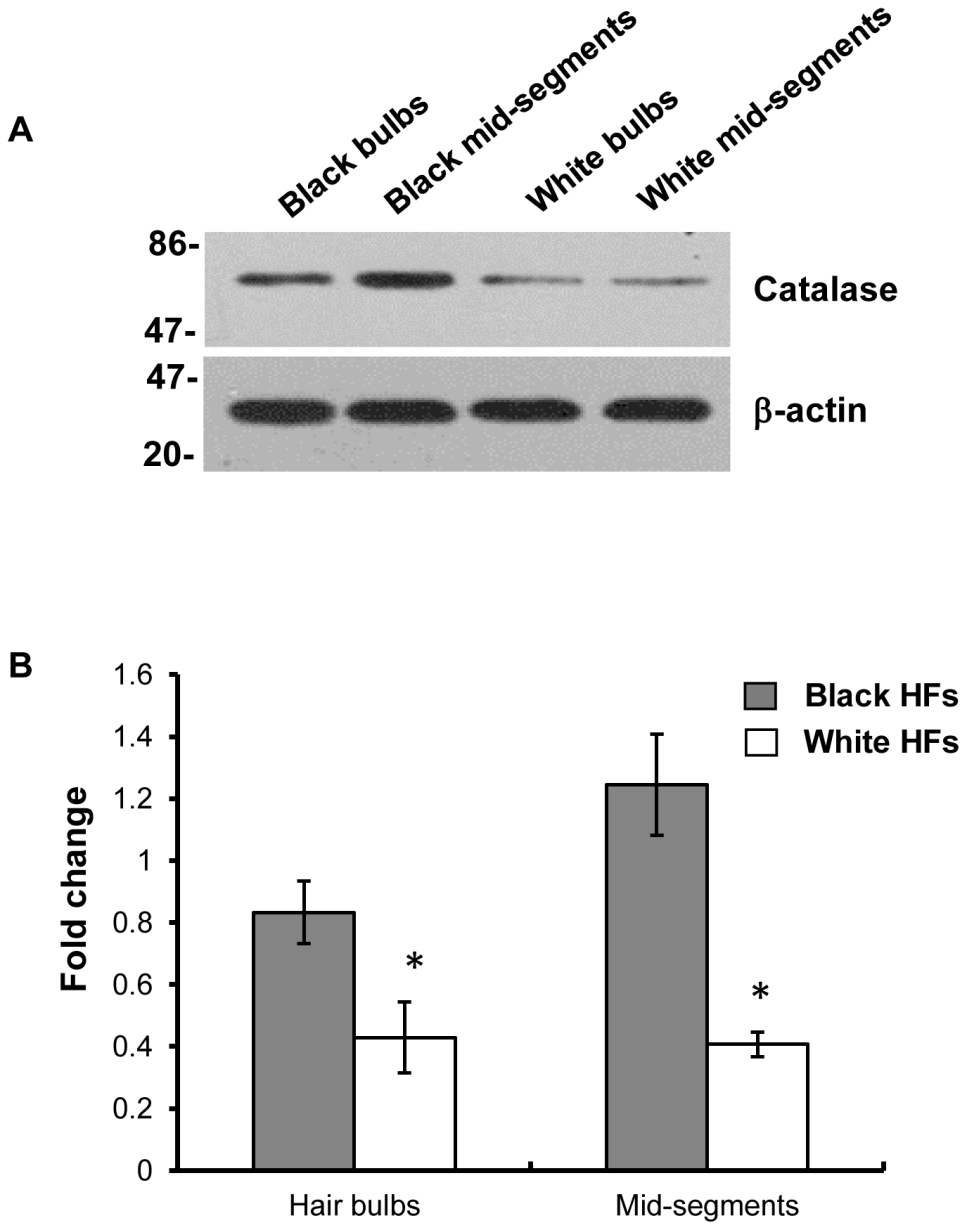
Expression level of catalase protein in hair follicles determined by western blotting. Equal amounts (15 μg per lane) of each protein extract were resolved using 10% SDS-PAGE electrophoresis. Protein loading variations were determined by immunoblotting with an anti-β-actin antibody. Representative blots are shown (A). The histogram (B) shows the densitometric quantification of data with means ± SD of 3 independent experiments, *P<0.05, compared to pigmented hair follicles.

**Figure 4:**
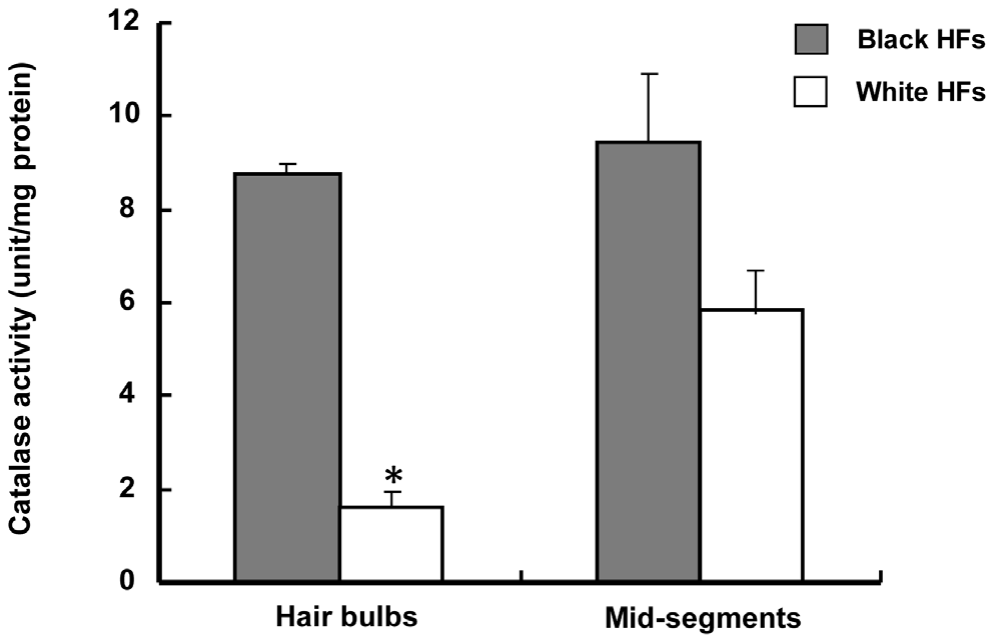
Catalase enzyme activity in hair follicles estimated by spectrophotometric assay. Catalase activity was determined spectrophotometrically using a commercial catalase analysis kit, as described in the text. Activities (unit per mg tissue protein) are expressed as means ± SD of 3 independent experiments, *P<0.05, compared to pigmented hair follicles.

Based on the above observations, we further proposed that an intrinsic deficiency of catalase protein might be a major cause of the oxidative damage of melanocytes in gray hair. Since mRNA expression levels are not always consistent with protein levels [24], we verified catalase protein levels in the hair bulbs and mid-segments of unpigmented hair follicles using western blotting and determined its catalytic activity using a spectrophotometric assay. Figures 3 and 4 show that levels of catalase protein expression and catalytic activity were significantly decreased in hair bulbs and mid-segments of unpigmented hair follicles compared with those areas of pigmented hair follicles. These results reveal that compromised catalase activity may contribute to the pathogenesis of premature graying hair.

### 3. Reduced Scavenging Activities against Hydroxyl Free Radicals in Hair Bulbs and in Mid-segments of Hair Follicles

**Figure 5:**
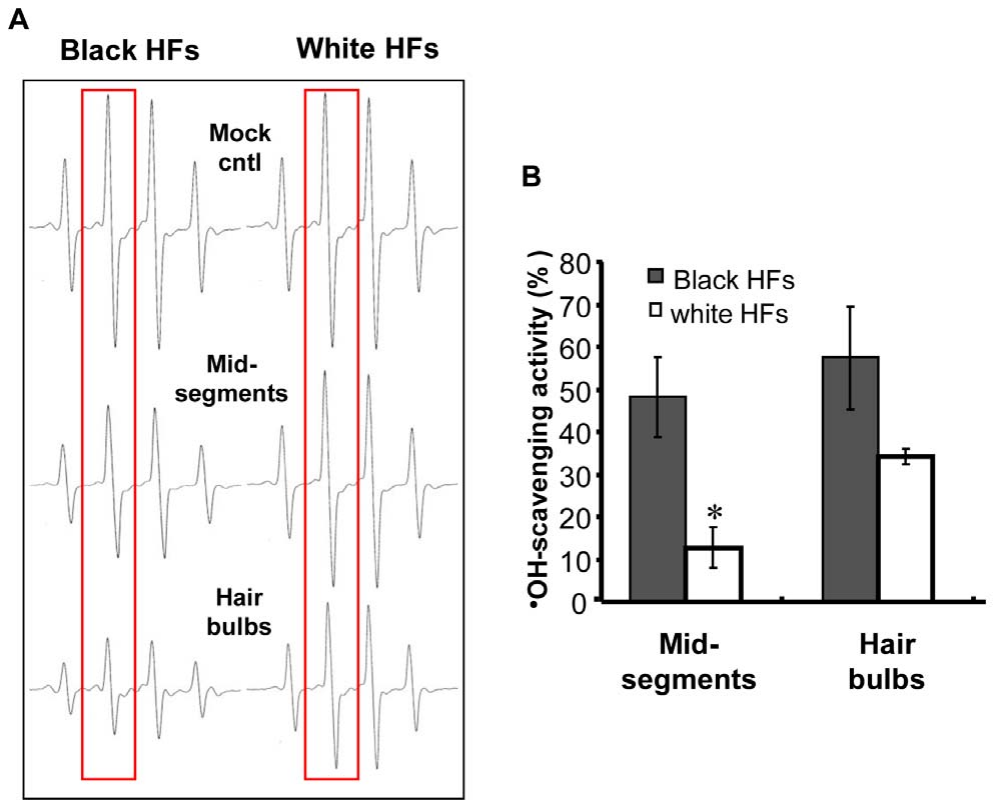
Hydroxyl radical-scavenging activities in hair follicles determined by an electron spin trapping ESR assay. The effects of equal amounts of each minced tissue sample on hydroxyl radical (⋅OH) generation in the Fenton reaction was studied using the ESR method, as described in the text. (A) Representative ESR spectra of DMPO-⋅OH with the hair bulb and mid-segment tissue samples. Hydroxyl radicals were generated by the Fenton reaction (DMPO: 400 mM). (B) Histogram showing hydroxyl radical-scavenging activity, which are expressed as means ± SD of 3 independent experiments, *P<0.05, compared to pigmented hair follicles.

A high concentration of H_2_O_2_ accumulates in graying hair follicles, as described in published reports [5], [6], which might be explained in part by the intrinsic deficiency of catalase in gray hair since catalase is an important antioxidant enzyme that catalyzes the conversion of H_2_O_2_ to water and molecular oxygen [6]. We also determined the non-specific hydroxyl radical scavenging activities of graying hair follicles in a Fenton reaction system using a spin trapping ESR assay. As shown in Figure 5, the hydroxyl radical-scavenging activities of hair bulbs and mid-segments of unpigmented hair follicles were significantly reduced compared with those areas of pigmented hair follicles, and are more prominent in the mid-segments (*P*<0.01). These results indicate that the loss of antioxidative activities in graying hair bulbs and bulge regions contributes to the abnormal accumulation of hydroxyl free radicals.

## Discussion

The regulation of anagen-coupled melanogenesis in human hair follicles has been enigmatic. Recently, a plethora of genes which play roles in either hair cycling growth or hair pigmentation has been identified by constructing transgenic mice [25], [26]. Despite the fact that incomplete melanocyte stem cell maintenance in the bulge region causes a hair graying phenotype in mice [27], as far as we know, characteristic premature hair graying has not been reported in wild-type C57 mice [28]. It seems plausible that data harvested from mouse models cannot fully explain the disappearance of functional melanocytes seen in gray hair bulbs of human anagen scalp hair follicles. We further propose that immature melanocyte precursor cells in the bulge region may also be destroyed along with hair bulb melanocytes, which has a critical implication for restoring pigment to gray hair in the clinical setting [29], [30]. Herein, we clearly provide qRT-PCR array results that indicate that genes encoding melanogenesis-related genes (TYR, TYRP1, MITF, PAX3, POMC, KIT, SOX10) in unpigmented hair bulbs are reduced more than 20-fold compared with pigmented hair bulbs. Meanwhile, marker genes of melanocyte precursor cells (PAX3, SOX10, DCT) were markedly decreased in unpigmented mid-segments compared with their pigmented analogues. These results demonstrate that melanocyte precursor cells in the bulge region are affected in graying hair follicles. More recently, Ito *et al*. noticed that over-expression of Wnt protein in mice potentiates hair neogenesis following wounding, but the new hair was unpigmented. This raised the possibility that differently coordinated activation of the Wnt pathway was required to modulate repopulation of keratinocyte stem cells and melanocyte stem cells in such regenerated follicles [31]. Our results reveal that significant down-regulation of the KRT15 and KRT19 genes is also detected in unpigmented mid-segments, which may represent the activation of quiescent keratinocyte stem cells in the bulge region [22], which in turn promotes the hair shaft growth of unpigmented hair [32].

Another focus of this study was the analysis of the antioxidative properties of isolated gray hair follicles. Those results show that hair bulbs and mid-segments of pigmented hair follicles express significant amounts of catalase protein and activity, whereas the level of catalase expression is significantly suppressed in unpigmented hair follicles. Our ESR data also reinforce the conclusion that compromised antioxidative activities in graying hair bulbs and bulge regions contribute to the abnormal accumulation of hydroxyl free radicals and the resulting oxidative destruction of hair follicle melanocytes.

Taken together, our findings add new understanding to whether immature melanocyte precursor cells in the bulge region of gray hair follicles are destroyed along with mature hair bulb melanocytes. They also reveal that an intrinsic deficiency of catalase protein may contribute to the abnormal accumulation of hydroxyl free radicals in gray hair follicles. In the future, it will be interesting to induce the targeted differentiation of bulge neural crest-derived stem cells into functional melanocytes in order to restore pigment to gray hair [33].
